# Association of Marital Status With Total and Cause-Specific Mortality in Asia

**DOI:** 10.1001/jamanetworkopen.2022.14181

**Published:** 2022-05-31

**Authors:** Chi Yan Leung, Hsi-Lan Huang, Sarah Krull Abe, Eiko Saito, Md. Rashedul Islam, Md. Shafiur Rahman, Ai Ikeda, Norie Sawada, Akiko Tamakoshi, Yu-Tang Gao, Woon-Puay Koh, Xiao-Ou Shu, Ritsu Sakata, Ichiro Tsuji, Jeongseon Kim, Sue K. Park, Chisato Nagata, San-Lin You, Jian-Min Yuan, Myung-Hee Shin, Wen-Harn Pan, Shoichiro Tsugane, Takashi Kimura, Wanqing Wen, Hui Cai, Kotaro Ozasa, Sanae Matsuyama, Seiki Kanemura, Yumi Sugawara, Aesun Shin, Keiko Wada, Chien-Jen Chen, Renwei Wang, Yoon-Ok Ahn, Habibul Ahsan, Paolo Boffetta, Kee Seng Chia, Keitaro Matsuo, You-Lin Qiao, Nathaniel Rothman, Wei Zheng, Daehee Kang, Manami Inoue

**Affiliations:** 1 Division of Prevention, National Cancer Center Institute for Cancer Control, Tokyo, Japan; 2 Institute for Global Health Policy Research, National Center for Global Health and Medicine, Tokyo, Japan; 3 Department of Global Health Policy, Graduate School of Medicine, The University of Tokyo, Tokyo, Japan; 4 Research Center for Child Mental Development, Hamamatsu University School of Medicine, Hamamatsu, Japan; 5 Juntendo University, School of Medicine, Department of Public Health, Tokyo, Japan; 6 Division of Cohort Research, National Cancer Center Institute for Cancer Control, Tokyo, Japan; 7 Department of Public Health, Hokkaido University Faculty of Medicine, Sapporo, Japan; 8 Department of Epidemiology, Shanghai Cancer Institute, Shanghai, China; 9 Healthy Longevity Translational Research Programme, Yong Loo Lin School of Medicine, National University of Singapore, Singapore; 10 Singapore Institute for Clinical Sciences, Agency for Science Technology and Research (A*STAR), Singapore; 11 Division of Epidemiology, Vanderbilt-Ingram Cancer Center, Vanderbilt Epidemiology Center, Vanderbilt University Medical Center, Nashville, Tennessee; 12 Radiation Effects Research Foundation, Hiroshima, Japan; 13 Tohoku University Graduate School of Medicine, Sendai, Japan; 14 Graduate School of Science and Policy, National Cancer Center, Gyeonggi-do, Republic of Korea; 15 Department of Preventive Medicine, Seoul National University College of Medicine, Seoul, Republic of Korea; 16 Department of Epidemiology and Preventive Medicine, Gifu University Graduate School of Medicine, Gifu, Japan; 17 School of Medicine and Big Data Research Center, Fu Jen Catholic University, New Taipei City, Taiwan; 18 Division of Cancer Control and Population Sciences, UPMC Hillman Cancer Center, University of Pittsburgh, Pittsburgh, Pennsylvania; 19 Department of Epidemiology, Graduate School of Public Health, University of Pittsburgh, Pittsburgh, Pennsylvania; 20 Department of Social and Preventive Medicine, Sungkyunkwan University School of Medicine, Seoul, Republic of Korea; 21 Institute of Biomedical Sciences, Academia Sinica, Taipei, Taiwan; 22 Institute of Population Health Sciences, National Health Research Institutes, Miaoli County, Taiwan; 23 Cancer Research Institute, Seoul National University, Seoul, Republic of Korea; 24 Genomics Research Center, Academia Sinica, Taipei, Taiwan; 25 Department of Public Health Sciences, University of Chicago, Chicago, Illinois; 26 Stony Brook Cancer Center, Stony Brook University, Stony Brook, New York; 27 Department of Medical and Surgical Sciences, University of Bologna, Bologna, Italy; 28 Saw Swee Hock School of Public Health, National University of Singapore, Singapore; 29 Division Cancer Epidemiology and Prevention, Aichi Cancer Center Research Institute, Nagoya, Japan; 30 Department of Cancer Epidemiology, Nagoya University Graduate School of Medicine, Nagoya, Japan; 31 Center for Global Health, School of Population Medicine and Public Health, Chinese Academy of Medical Sciences and Peking Union Medical College, Beijing, China; 32 Division of Cancer Epidemiology and Genetics, Occupational and Environmental Epidemiology Branch, National Cancer Institute, Bethesda, Maryland; 33 College of Medicine, Seoul National University, Seoul, Republic of Korea; 34 Department of Cancer Epidemiology, Graduate School of Medicine, The University of Tokyo, Tokyo, Japan

## Abstract

**Importance:**

Marital status has been shown to be associated with mortality, but evidence in Asian populations is limited.

**Objective:**

To examine the association of marital status with total and cause-specific mortality.

**Design, Setting, and Participants:**

This cohort study included individual participant data from 16 prospective studies in the Asia Cohort Consortium conducted between 1963 and 2015. Asian participants with complete information on marital and vital status were included. Study-specific hazard ratios (HRs) and 95% CIs were estimated using Cox proportional hazards model and then pooled using a random-effects meta-analysis. The analysis began in February 2021 and ended in August 2021.

**Exposures:**

Marital status.

**Main Outcomes and Measures:**

All-cause and cause-specific mortality.

**Results:**

Of 623 140 participants (326 397 women [52.4%] and 296 743 men [47.6%]; mean [SD] age, 53.7 [10.2] years; mean [SD] follow-up time, 15.5 [6.1] years), 123 264 deaths were ascertained. Compared with married individuals, those who were unmarried had pooled HRs of 1.15 (95% CI, 1.07-1.24) for total mortality, 1.12 (95% CI, 1.03-1.22) for cerebrovascular disease mortality, 1.20 (95% CI, 1.09-1.31) for coronary heart disease mortality, 1.17 (95% CI, 1.07-1.28) for circulatory system diseases mortality, 1.06 (95% CI, 1.01-1.11) for cancer mortality, 1.14 (95% CI, 1.05-1.23) for respiratory diseases mortality, and 1.19 (95% CI, 1.05-1.34) for external causes of death. Positive associations with total mortality were also observed for those who were single (HR, 1.62; 95% CI, 1.41-1.86), separated (HR, 1.35; 95% CI, 1.13-1.61), divorced (HR, 1.38; 95% CI, 1.13-1.69), and widowed (HR, 1.09; 95% CI, 1.04-1.13). In subgroup analyses, the positive association persisted across baseline health conditions, and the risk of death was more pronounced among men or people younger than 65 years.

**Conclusions and Relevance:**

This large pooled cohort study of individual participant data provides strong evidence that being unmarried, as well as belonging to the unmarried subcategories, was positively associated with total and cause-specific mortality. Investment of targeted social support services might need to be considered in light of the mortality differences between married and unmarried individuals.

## Key Points

**Question:**

What is the association between marital status and mortality outcomes in Asian populations?

**Findings:**

In this cohort study of 623 140 individuals from 16 prospective cohorts participating in the Asia Cohort Consortium, a pooled analysis found that being unmarried was associated with a higher risk of all-cause and cause-specific mortality, compared with being married. The excess risks of death persisted across health conditions and were particularly pronounced among men and people younger than 65 years.

**Meaning:**

These findings highlight that being unmarried substantially increases the risk of death among Asian individuals.

## Teaser

This cohort study examines the association of marital status with total and cause-specific mortality in Asian populations.

## Introduction

Globally, the marriage rate has decreased over the last decades.^1^ There is broad agreement on the association of marital status with mortality.^2,3,4^ A recent meta-analysis^3^ showed that individuals who were unmarried had a higher risk of all-cause and cause-specific mortality than their married counterparts. Achieving socioeconomic support and health-promoting behaviors have been implicated to be the factors contributing to these protective associations of marriage.^5,6,7,8,9^ Physiologically, a higher level of cortisol and a flatter diurnal cortisol slope are found in unmarried individuals,^10^ which are linked to an increased risk of carotid atherosclerosis,^11^ poor glycemic control in patients with diabetes,^12^ metabolic syndrome,^13^ and shorter survival time in patients with cancer.^14,15^

Although the pattern of marriage differs among societies, few studies have reported the linkage between marriage and mortality in non-Western populations.^16,17,18,19,20^ Marriages in East Asia have distinct features, including a strong tradition of extended family coresidence, an emphasis on family lineage that stabilizes the institution of marriage, and strong family ties,^21^ which have been associated with lower mortality risk.^22^ In addition, the pronounced marital selection outcomes and financial burden of single-earner households in Asian society have been suggested as factors associated with higher mortality among single Asian individuals.^23^ Together, we hypothesized that being married would be associated with survival benefits in Asian populations. Nevertheless, previous studies^16,17,18,19,20^ of Asian cohorts have published inconsistent results. It is also noteworthy that, in previous investigations, there were concerns regarding the potential effects of reverse causation, where initial presentation of undiagnosed diseases may influence the likelihood of marriage dissolution.^24^ In addition, the association may differ across participant subgroups. Prior studies investigating potential modifying effects of age and sex demonstrated mixed results.^3,25,26,27,28,29^ With most studies focused on the generally healthy population,^3^ less is known regarding the mortality differences by marital status among individuals with chronic diseases. Given the increasing prevalence of noncommunicable diseases in Asian countries,^30^ it is imperative to understand whether the association between marital status and mortality differs among people with underlying comorbidity. We therefore conducted a cohort study using individual-level data from the Asia Cohort Consortium (ACC), with more than 1 million participants, to ascertain the association between marital status and cause-specific mortality.

## Methods

### Study Population

The ACC was established to foster collaborative epidemiological research in Asian countries using pooled multicenter cohort data. The ACC currently contains individual-level data from more than 1 million participants. Of 23 prospective cohort studies in the ACC, we used data from 16 cohorts with available data on marital status. The study designs of the included cohorts have been described elsewhere.^31^ Participating cohorts from 5 countries were included: Mainland China, Shanghai Cohort Study, Shanghai Men’s Health Study, and Shanghai Women’s Health Study; Japan, Japan Collaborative Cohort Study, Japan Public Health Center-Based Prospective Study, Miyagi Cohort Study, Ohsaki National Health Insurance Cohort Study, Life Span Study Cohort–Radiation Effects Research Foundation, and Takayama Study; Republic of Korea, Korea Multi-Center Cancer Cohort Study, Korea National Cancer Center Cohort, and Seoul Male Cancer Cohort; Singapore, Singapore Chinese Health Study; and Taiwan, Community-Based Cancer Screening Project and Cardiovascular Disease Risk Factor Two-Township Study. Of the 681 533 potential participants, we excluded those with missing information on marital status (37 600 individuals) or vital status (20 793 individuals). This study was approved by the ethics committees of each cohort study and by the institutional review board of the ACC coordinating center. Informed consent (either written or oral, depending on the cohort) was obtained from all participants per study cohort. This study follows the Strengthening the Reporting of Observational Studies in Epidemiology (STROBE) reporting guideline.

### Assessment of Exposure and Other Covariates

Cohort questionnaires at baseline assessed information on lifestyle factors, sociodemographic characteristics, diet, and medical history of type 2 diabetes, hypertension, cancer, coronary heart disease, and cerebrovascular disease. Among 16 cohorts included, participants from Korea National Cancer Center Cohort, Korea Multi-Center Cancer Cohort Study, Community-Based Cancer Screening Project, and Cardiovascular Disease Risk Factor Two-Township Study were interviewed by trained interviewers, whereas the other cohorts used self-administered questionnaires. Brief descriptions of participating cohorts are presented in eTable 1 in the Supplement. Information on marital status from each cohort was harmonized and classified into 5 categories: married, single, separated, widowed, and divorced. Analyses for subcategories of unmarried status (single, separated, widowed, and divorced) were performed where data were available (eAppendix in the Supplement).

### Outcome Ascertainment

Our primary end point was death from all causes. Deaths were ascertained via linkage to the death certificates or active follow-up. The causes of death were classified using the *International Classification of Diseases, Ninth Revision* or *International Classification of Diseases, Tenth Revision* (eAppendix in the Supplement).

### Statistical Analysis

In this study, we performed a 2-stage meta-analysis of individual participant data to estimate the pooled hazard ratios (HRs) and 95% CIs.^32^ First, a Cox proportional hazards regression model with age as the time scale was used to estimate HRs and 95% CIs across marital status categories by cohorts. Married individuals were the reference group. Time of entry was age at the baseline interview, and exit time was age at death or last follow-up, whichever came first. The multivariable model was adjusted for potential confounders, including sex, age at baseline (year, continuous), education (no formal education or primary education, secondary education, trade or technical education, university education or above, and missing), smoking status (never, former, current, and missing), alcohol intake (nondrinker, drinker, and missing), physical activity (none or almost none, low, intermediate, high, and missing), and baseline health conditions (cerebrovascular disease, coronary heart disease, cancer, hypertension, and diabetes). We used missing indicator variables to code missing values for potential confounders. Second, we obtained the overall estimates using random-effects meta-analysis. To minimize the bias due to reverse causation, we repeated the analyses and excluded deaths occurring within the first 5 years of follow-up.

We performed prespecified stratified analysis according to age at baseline (<65 and ≥65 years), sex (men and women), health status (prior diagnosis of cancer, cerebrovascular disease, or coronary heart disease; prior diagnosis of type 2 diabetes or hypertension but not cancer, cerebrovascular disease, or coronary heart disease; and healthy participants with none of the aforementioned diseases), birth year (before and after 1940), and country. Metaregression was used to test for effect modification. We used Stata statistical software version 16.0 (StataCorp) for analysis. All reported *P* values were 2-sided, and *P* < .05 was considered to be significant. Data analysis was performed from February to August 2021.

## Results

A total of 623 140 participants were included in the analyses: 296 743 men (47.6%) and 326 397 women (52.4%). The mean (SD) age at baseline was 53.7 (10.2) years. During a mean (SD) follow-up of 15.5 (6.1) years, 123 264 deaths were ascertained, of which 37 394 were classified as death from circulatory system diseases, 8013 were from coronary heart disease, 14 563 were from cerebrovascular disease, 41 362 were from cancer, 13 583 were from respiratory diseases, and 7795 were from external causes. The mean prevalence of being married was 86.4% (538 377 individuals), ranging from 76.4% (36 046 individuals) in the Life Span Study Cohort–Radiation Effects Research Foundation to 98.7% (13 777 individuals) in the Seoul Male Cancer Cohort. Baseline characteristics of participants of each cohort are shown in Table 1 and eTable 2 in the Supplement.

**Table 1.** Characteristics of Participating Cohorts in the Asia Cohort Consortium

Country and cohort	Follow-up duration, mean (SD), y	Years of study entry	Participants, No. (%)		Age at entry, mean (SD), y	Married individuals, No. (%)	Cause of death, No. of individuals				
			Men	Women			All-cause	Circulatory system diseases	Cancer	Respiratory diseases	External
Mainland China											
SCS (n = 16 751)	22.4 (8.2)	1986-1989	16 751 (100.0)	0	55.9 (5.7)	15 853 (94.6)	10 181	3768	3533	1210	269
SMHS (n = 53 205)	9.5 (1.7)	2001-2006	53 205 (100.0)	0	55.7 (9.7)	51 761 (97.3)	4558	1487	2020	331	104
SWHS (n = 74 743)	14.9 (2.2)	1996-2000	0	74 743 (100.0)	52.6 (9.1)	66 345 (88.8)	7458	2432	3134	303	207
Japan											
JACC (n = 74 989)	12.8 (3.2)	1988-1990	31 337 (41.8)	43 652 (58.2)	57.1 (10.0)	65 829 (87.8)	10 270	3096	3842	1157	733
JPHC1 (n = 42 587)	21.1 (4.2)	1990-1992	20 352 (47.8)	22 235 (52.2)	49.6 (6.0)	33 042 (77.6)	7324	1807	2881	596	683
JPHC2 (n = 55 841)	17.8 (4.0)	1992-1995	26 407 (47.3)	29 434 (52.7)	54.2 (8.8)	47 112 (84.4)	12 505	3150	4608	1436	846
Miyagi (n = 37 921)	22.5 (4.8)	1990	18 919 (49.9)	19 002 (50.1)	51.9 (7.5)	34 259 (90.3)	11 549	2184	2809	862	2494
Ohsaki (n = 26 950)	11.7 (3.2)	1995	14 609 (54.2)	12 341 (45.8)	58.0 (10.8)	22 365 (83.0)	7435	2177	2415	1007	203
RERF (n = 47 200)	22.0 (10.2)	1963-1993	18 691 (39.6)	28 509 (60.4)	52.1 (13.6)	36 046 (76.4)	24 400	9007	6619	3210	667
Takayama (n = 30 574)	13.8 (3.8)	1992	13 961 (45.7)	16 613 (54.3)	55.8 (12.8)	25 076 (82.0)	5818	1930	1698	810	372
Republic of Korea											
KMCC (n = 19 337)	13.9 (4.5)	1993-2004	7718 (39.9)	11 619 (60.1)	53.9 (14.5)	14 920 (77.2)	3577	835	1108	317	357
KNCC (n = 37 638)	9.3 (3.3)	2001-2015	19 113 (50.8)	18 525 (49.2)	49.8 (9.2)	33 946 (90.2)	553	48	207	6	57
Seoul-Male (n = 13 957)	15.6 (1.8)	1992-1993	13 957 (100.0)	0	49.2 (5.2)	13 777 (98.7)	901	150	498	29	101
Singapore, SCHS/SGC (n = 62 658)	14.0 (3.6)	1993-1999	27 593 (44.0)	35 065 (56.0)	56.4 (8.0)	52 177 (83.3)	13 238	4500	4715	2033	361
Taiwan											
CBCSP (n = 23 670)	15.3 (2.5)	1991-1992	11 883 (50.2)	11 787 (49.8)	47.3 (10.0)	21 771 (92.0)	2688	537	994	160	276
CVDFACTS (n = 5119)	15.0 (2.9)	1990-1993	2247 (43.9)	2872 (56.1)	47.5 (15.6)	4098 (80.1)	809	213	214	83	55
Total (N = 623 140)	15.5 (6.1)	1963-2015	296 743 (47.6)	326 397 (52.4)	53.7 (10.2)	538 377 (86.4)	123 264	37 321	41 295	13 550	7785

Abbreviations: CBCSP, Community-based Cancer Screening Project; CVDFACTS, Cardiovascular Diseases Risk Factor Two-Township Study; JACC, Japan Collaborative Cohort Study; JPHC, Japan Public Health Center-based prospective Study; KMCC, Korean Multi-center Cancer Cohort Study; KNCC, Korea National Cancer Center Cohort; Miyagi, Miyagi Cohort Study; Ohsaki, Ohsaki National Health Insurance Cohort Study; RERF, Life Span Study Cohort–Radiation Effects Research Foundation; SCHS/SGC, Singapore Chinese Health Study; SCS, Shanghai Cohort Study; Seoul-Male, Seoul Male Cancer Cohort; SMHS, Shanghai Men’s Health Study; SWHS, Shanghai Women’s Health Study; Takayama, Takayama Study.

### Marital Status and Total Mortality

In multivariable analyses, being unmarried was associated with a 15% greater risk of all-cause mortality (pooled multivariable HR, 1.15; 95% CI, 1.07-1.24) compared with being married (Figure and eTable 3 in the Supplement). We further examined associations of subcategories of unmarried status with mortality. Compared with married participants, positive associations for death from all causes were found among those who were single (pooled HR, 1.62; 95% CI, 1.41-1.86), separated (pooled HR, 1.35; 95% CI, 1.13-1.61), divorced (pooled HR, 1.38; 95% CI, 1.13-1.69), and widowed (pooled HR, 1.09; 95% CI, 1.04-1.13). The associations remained positive when we excluded participants who died during the first 5 years of follow-up (Figure and eTable 3 in the Supplement).

**Figure.**
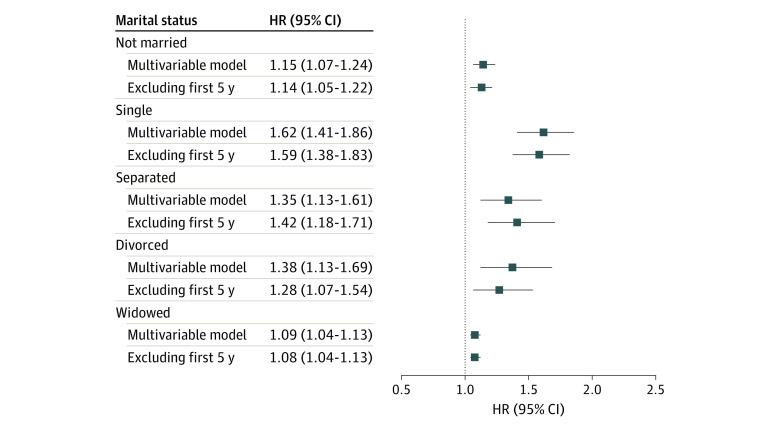
Association of Marital Status With All-Cause Mortality Compared With Married Individuals in Asian Populations Multivariable Cox regression model was adjusted for sex, age at baseline (year, continuous), smoking status (never, former, current, and missing), education (no formal education or primary education, secondary education, trade or technical education, university education or above, and missing), physical activity (none or almost none, low, intermediate, high, and missing), alcohol intake (nondrinker, drinker, and missing), and baseline health conditions (cerebrovascular disease, coronary heart disease, cancer, hypertension, and diabetes). HR indicates hazard ratio.

### Marital Status and Cause-Specific Mortality

The pooled multivariable HRs for unmarried compared with married individuals were 1.12 (95% CI, 1.03-1.22) for cerebrovascular disease mortality, 1.20 (95% CI, 1.09-1.31) for coronary heart disease mortality, 1.17 (95% CI, 1.07-1.28) for circulatory system diseases mortality, 1.06 (95% CI, 1.01-1.11) for cancer mortality, 1.14 (95% CI, 1.05-1.23) for respiratory diseases mortality, and 1.19 (95% CI, 1.05-1.34) for external causes of death (Table 2). Compared with those who were married, being single was associated with a greater risk of death from cerebrovascular disease, coronary heart disease, circulatory system diseases, cancer, respiratory diseases, and external causes (Table 2). Being widowed was associated with higher risks of death from cerebrovascular disease, coronary heart disease, circulatory system diseases, and external causes. There were higher risks of coronary heart disease mortality and external causes of death among separated participants. Being divorced was positively associated with risk of death from cerebrovascular disease, with similar magnitude of associations observed with risk of death from circulatory system diseases, respiratory diseases, and external causes (Table 2). Similar patterns were observed after the exclusion of mortality cases with the first 5 years of follow-up.

**Table 2.** Association of Marital Status With Risk of Cause-Specific Mortality in Asian Populations

Model and cause of death	Married (n = 538 377)	Not married (n = 84 763)	Single (n = 13 159)	Separated (n = 1168)	Divorced (n = 4671)	Widowed (n = 33 997)
Multivariable model						
Cerebrovascular disease						
Deaths, No.	11 699	2864	339	18	99	1688
HR (95% CI)^a^	1 [Reference]	1.12 (1.03-1.22)	1.59 (1.22-2.06)	1.01 (0.63-1.60)	1.45 (1.18-1.79)	1.08 (1.00-1.17)
Coronary heart disease						
Deaths, No.	6461	1552	188	15	49	882
HR (95% CI)^a^	1 [Reference]	1.20 (1.09-1.31)	1.72 (1.31-2.27)	2.21 (1.32-3.71)	1.40 (0.85-2.30)	1.13 (1.01-1.26)
Circulatory system diseases						
Deaths, No.	29 721	7673	937	49	212	4348
HR (95% CI)^a^	1 [Reference]	1.17 (1.07-1.28)	1.68 (1.37-2.07)	1.31 (0.98-1.74)	1.56 (1.12-2.17)	1.11 (1.04-1.17)
Cancer						
Deaths, No.	34 692	6670	877	55	213	3179
HR (95% CI)^a^	1 [Reference]	1.06 (1.01-1.11)	1.31 (1.17-1.45)	1.25 (0.96-1.64)	1.10 (0.93-1.30)	1.04 (0.99-1.08)
Respiratory diseases						
Deaths, No.	10 845	2738	362	12	63	1429
HR (95% CI)^a^	1 [Reference]	1.14 (1.05-1.23)	1.80 (1.39-2.35)	2.28 (0.82-6.37)	1.42 (1.11-1.83)	1.05 (0.98-1.13)
External causes of death						
Deaths, No.	6565	1230	217	12	45	403
HR (95% CI)^a^	1 [Reference]	1.19 (1.05-1.34)	1.63 (1.28-2.06)	2.09 (1.18-3.71)	1.56 (1.16-2.11)	1.12 (1.00-1.25)
Excluding first 5 y of follow-up						
Cerebrovascular disease						
Deaths, No.	9905	2328	277	15	83	1374
HR (95% CI)^a^	1 [Reference]	1.10 (1.01-1.21)	1.47 (1.11-1.95)	1.16 (0.70-1.94)	1.47 (1.18-1.83)	1.07 (0.99-1.16)
Coronary heart disease						
Deaths, No.	5491	1255	154	13	40	697
HR (95% CI)^a^	1 [Reference]	1.16 (1.05-1.28)	1.76 (1.29-2.40)	2.35 (1.35-4.10)	1.35 (0.78-2.32)	1.09 (0.99-1.22)
Circulatory system diseases						
Deaths, No.	25 414	6342	788	41	165	3529
HR (95% CI)^a^	1 [Reference]	1.15 (1.05-1.26)	1.67 (1.35-2.06)	1.41 (1.03-1.95)	1.42 (1.03-1.96)	1.09 (1.03-1.15)
Cancer						
Deaths, No.	28 822	5449	707	44	159	2522
HR (95% CI)^a^	1 [Reference]	1.05 (0.99-1.12)	1.27 (1.12-1.43)	1.27 (0.95-1.72)	1.04 (0.87-1.24)	1.04 (1.00-1.09)
Respiratory diseases						
Deaths, No.	9664	2415	319	11	55	1240
HR (95% CI)^a^	1 [Reference]	1.15 (1.05-1.25)	1.72 (1.31-2.26)	2.39 (1.12-5.14)	1.47 (1.12-1.93)	1.08 (0.99-1.18)
External causes of death						
Deaths, No.	5631	1006	177	10	35	292
HR (95% CI)^a^	1 [Reference]	1.16 (1.03-1.29)	1.52 (1.25-1.86)	2.76 (1.47-5.18)	1.75 (1.15-2.67)	1.08 (0.95-1.24)

Abbreviation: HR, hazard ratio.

^a^ Multivariable Cox regression model was adjusted for sex, age at baseline (year, continuous), smoking status (never, former, current, and missing), education (no formal education or primary education, secondary education, trade or technical education, university education or above, and missing), physical activity (none or almost none, low, intermediate, high, and missing), alcohol intake (nondrinker, drinker, and missing), and baseline health conditions (cerebrovascular disease, coronary heart disease, cancer, hypertension, and diabetes).

### Subgroup Analyses

We further evaluated the association between marital status and mortality risk stratified by health status at baseline (Table 3). In subgroup analyses defined by variables (e.g. disease status at baseline), there were positive associations between unmarried and total mortality in each subgroup (Table 3). An insignificant *P* value for interaction indicated that there was no difference between 3 groups: (1) cancer, coronary heart disease, or cerebrovascular disease; (2) diabetes or hypertension only; and (3) healthy. We found that being single was associated with higher risk of mortality among healthy participants than among those with a history of cancer, cerebrovascular disease, or coronary heart disease (*P* for interaction = .01). The association for the separated was also modified by health status (*P* for interaction = .01). In subgroup analyses, we found that the associations between being unmarried, widowed, or divorced and risk of death from all causes were modified by sex (Table 3). Positive associations were found among unmarried (*P* for interaction = .003), widowed (*P* for interaction =. 049), and divorced (*P* for interaction = .047) men, but no association was found among women. The associations between marital status (unmarried or single) and risk of total mortality were modified by age (*P* for interaction < .001) (Table 3). The HRs were higher among single participants younger than 65 years (HR, 1.79; 95% CI, 1.54-2.08) than those who were aged 65 years and older (HR, 1.11; 95% CI, 1.02-1.22) (*P* for interaction < .001). Subgroup analyses according to birth year and country are presented in eTable 4 in the Supplement.

**Table 3.** All-Cause Mortality Associated With Marital Status Stratified by Individual Characteristics in Asian Populations

Variable	Married	Not married		Single		Separated		Divorced		Widowed	
		HR (95% CI)	*P* for interaction	HR (95% CI)	*P* for interaction	HR (95% CI)	*P* for interaction	HR (95% CI)	*P* for interaction	HR (95% CI)	*P* for interaction
Disease status at baseline^a^											
Cancer, coronary heart disease, or cerebrovascular disease	1 [Reference]	1.10 (1.05-1.16)	.26	1.28 (1.10-1.49)	.01	1.91 (1.33-2.74)	.01	1.45 (0.98-2.16)	.42	1.10 (1.03-1.18)	.62
Deaths, No.	9132	2316		178		30		79		1440	
Diabetes or hypertension only	1 [Reference]	1.10 (1.02-1.19)		1.39 (1.18-1.63)		2.03 (1.25-3.29)		1.20 (1.02-1.42)		1.10 (1.04-1.16)	
Deaths, No.	30 123	7078		784		42		153		3667	
Healthy^b^	1 [Reference]	1.19 (1.09-1.30)		1.77 (1.51-2.08)		1.18 (0.96-1.45)		1.51 (1.19-1.93)		1.09 (1.03-1.14)	
Deaths, No.	60 994	13 621		2110		92		409		6544	
Sex^c^											
Male	1 [Reference]	1.23 (1.12-1.35)	.003	1.67 (1.43-1.94)	.11	1.62 (1.23-2.13)	.09	1.48 (1.28-1.75)	.047	1.13 (1.06-1.21)	.049
Deaths, No.	63 594	9405		1799		51		355		3313	
Female	1 [Reference]	1.03 (0.97-1.10)		1.39 (1.18-1.62)		1.21 (1.00-1.46)		1.22 (0.91-1.62)		1.03 (0.97-1.10)	
Deaths, No.	36 655	13 610		1273		113		286		8338	
Age at baseline^d^											
<65 y	1 [Reference]	1.26 (1.14-1.38)	<.001	1.79 (1.54-2.08)	<.001	1.67 (1.25-2.22)	.05	1.53 (1.22-1.92)	.06	1.09 (1.03-1.15)	.19
Deaths, No.	68 812	12 182		2308		89		449		4235	
≥65 y	1 [Reference]	1.00 (0.94-1.07)		1.11 (1.02-1.22)		1.12 (0.89-1.40)		1.06 (0.84-1.35)		1.04 (0.99-1.09)	
Deaths, No.	31 437	10 833		764		75		192		7416	

Abbreviation: HR, hazard ratio.

^a^ Multivariable Cox regression model was adjusted for sex, age at baseline (year, continuous), smoking status (never, former, current, and missing), education (no formal education or primary education, secondary education, trade or technical education, university education or above, and missing), physical activity (none or almost none, low, intermediate, high, and missing), and alcohol intake (nondrinker, drinker, and missing).

^b^ Refers to participants who had no history of cancer, cerebrovascular disease, coronary heart disease, diabetes, or hypertension.

^c^ Multivariable Cox regression model was adjusted for age at baseline (year, continuous), smoking status (never, former, current, and missing), education (no formal education or primary education, secondary education, trade or technical education, university education or above, and missing), physical activity (none or almost none, low, intermediate, high, and missing), alcohol intake (nondrinker, drinker, and missing), and baseline health conditions (cerebrovascular disease, coronary heart disease, cancer, hypertension, and diabetes).

^d^ Multivariable Cox regression model was adjusted for sex, smoking status (never, former, current, and missing), education (no formal education or primary education, secondary education, trade or technical education, university education or above, and missing), physical activity (none or almost none, low, intermediate, high, and missing), alcohol intake (nondrinker, drinker, and missing), and baseline health conditions (cerebrovascular disease, coronary heart disease, cancer, hypertension, and diabetes).

## Discussion

In this cohort study using pooled data for 623 140 Asian individuals from 16 prospective cohorts, we demonstrated that unmarried individuals had a 15% increased risk of total mortality, after adjusting for potential confounders. Compared with married participants, those who were single, separated, widowed, and divorced had a greater risk of death from all causes. The results were similar after excluding deaths occurring within the first 5 years, which confirmed the robustness of our findings. This pooled analysis further showed that being unmarried was associated with a greater risk of death from 6 specific causes. The large sample size of the ACC allowed us to examine whether the association between marital status and mortality risks differed across subgroups of sex, age, and health condition at baseline.

Our results confirmed the findings in a recent meta-analysis,^3^ which reported pooled risk ratios (RRs) of 1.33 (95% CI, 1.24-1.43) for death from all causes, 1.37 (95% CI, 1.21-1.55) for cancer, and 1.11 (95% CI, 1.07-1.15) for circulatory system diseases for unmarried vs married individuals. The evidence for respiratory disease mortality has been sparse, however. In accordance with our findings, data from the Japan Collaborative Cohort Study^16^ and the National Cohort Study in Denmark^25^ showed a greater risk of death from respiratory diseases among those who were unmarried compared with married individuals.

Although the association between unmarried subcategories with mortality has been extensively investigated, the results are inconclusive.^2,3,4^ An early meta-analysis^2^ found that the positive association was greater among divorced or separated individuals (RR, 1.16; 95% CI, 1.09-1.23) than among widowed individuals (RR, 1.11; 95% CI, 1.08-1.14) or those who were never married (RR, 1.11; 95% CI, 1.07-1.15); however, only 5 of the 53 included studies were from Asian populations, and they were limited by small samples. More studies have been conducted since but with conflicting results (HR for single individuals, 1.54; 95% CI, 1.46-1.63; HR for divorced or separated individuals, 1.43; 95% CI, 1.34-1.52; HR for widowed individuals, 1.23; 95% CI, 1.19-1.26).^3^ In our study of 623 140 Asian individuals with a mean follow-up of 15.5 years, we found that, among all unmarried subcategories, being single was most associated with death from all causes. These findings could be explained by self-selection of single individuals with less favorable socioeconomic and psychosocial status to enter into marriage^33^ and by the disparity in social support across subgroups of unmarried individuals. The duration of living with a spouse may also play an important role in mortality risks, because individuals who were divorced, separated, or widowed had been cohabitating with a spouse for a time during marriage. Similar findings were seen for deaths from other major causes, except for coronary heart disease mortality and external causes of death, for which separation was associated with greater risks than other unmarried status. The observed associations may be a consequence of the reduction in the ability to cope with stress accrued from separation.^4^

Prior literature reported decreased survival for unmarried patients with known cardiovascular disease^4,24^ and cancer.^34^ Although marital status has been associated with the incidence of hypertension^35^ and type 2 diabetes,^36^ none of the previous studies has explored mortality outcomes by marital status after the onset of hypertension or diabetes, rendering interpretation of long-term outcomes difficult. To our knowledge, the current study is the first to examine the association between marital status and mortality risks among participants with various health statuses within the same cohort. Our results suggest that there is a survival advantage not only for married individuals with life-threatening diseases (cancer, coronary heart disease, and cerebrovascular disease) but also for those with diabetes or hypertension. The protective outcomes of marriage among people with chronic diseases may be associated with the encouragement from partners to seek medical help and adhere to treatment.^37,38^ Given the wealth of evidence indicating the positive association between marital status and mortality, the importance of adequate support to unmarried individuals with chronic diseases cannot be neglected.

The current analysis found a significant interaction between age and all-cause mortality among unmarried groups, especially for the single and the separated. Sorlie et al^26^ reported that unmarried participants younger than 65 years experienced a higher risk of mortality than those who were older. The smaller association in elderly populations may be due to extramarital support from relatives or community, diminished health-promoting outcomes of marriage, and reduction of social support disparity between married and unmarried groups.^2,37^ The exact reasons still need to be explored. Some previous studies found that sex is a modifier of the association of marriage with death,^27,28^ whereas others failed to demonstrate the disparity.^25,29^ One meta-analysis^3^ summarizing 3 studies from Asia and 12 studies from America and Europe reported a women-to-men relative RR of 0.86 (95% CI, 0.79-0.94) for total mortality associated with being unmarried. In our study, we found that being unmarried was associated with higher mortality among men but not among women, reflecting the possibility that, in Asian marriages, gender inequality in the division of domestic labor and high expectations of childcare for women^21^ may counteract the health benefits of marriage among women. Another possible explanation is that, compared with married women, a higher employment rate among unmarried women may have contributed to financial security and favorable health outcomes.^16,39^ Furthermore, unmarried men may receive less social support and financial protection from the government than unmarried women in Asia.^16,39^

### Strengths and Limitations

The strength of our pooled analyses includes the prospective design of each study, which minimized potential recall bias and selection bias. In addition, to our knowledge, the present study is the largest pooled analysis of Asian populations from 16 cohorts, which allowed us to provide estimates with sufficient precision and to examine reverse causation.

However, our study has limitations that merit further discussion. First, given the observational nature of the study and the lack of data on income, residual confounding could exist. Second, we excluded participants with missing information on marriage and vital status, and this may lead to selection bias if participants with complete information are not representative of the cohort population. Also, individuals with higher socioeconomic status may be more likely to participate in the study and less likely to be lost to follow-up. Third, information regarding cohabitation and changes in marital status was not captured in our data set, and misclassification is possible. However, later life marital dissolution may be more dominant than remarriage, which could diminish the observed associations. In addition, the rate of nonmarital cohabitation is likely to be small in the included countries of the present analysis.^21^ Such participants would be grouped as unmarried in the analyses and were expected to show better survival outcomes, which, in turn, biased the association toward the null. Fourth, the likelihood of alterations in marital status before the baseline survey may be influenced by initial symptoms of undiagnosed diseases. To minimize bias from reverse causation, we performed analysis excluding deaths within the first 5 years of follow-up. Similar results were observed, suggesting these biases could be minimal in our findings. Fifth, covariates were assessed at baseline; therefore, the changes in these variables over time were not considered. Although we have included 16 population-based cohorts in Asia, we acknowledge that the heterogeneity within the region may not be fully represented by this study. Sixth, the mean age of 53.7 years at baseline was young with regard to the outcome of mortality, despite the long follow-up of 15.5 years.

## Conclusions

In this large-scale cohort study pooling data from multiple cohorts, being unmarried was associated with a higher risk of total and cause-specific mortality. The association persisted across baseline health conditions and was particularly evident in men and participants who were younger than 65 years at baseline. Our findings underscore the potential to reduce unfavorable mortality outcomes among unmarried individuals in Asia.

## Notes

Supplement. **eAppendix.** Supplemental Methods **eTable 1.** Descriptions and Basic Characteristics of Participating Cohorts **eTable 2.** Baseline Characteristics for 623,140 Participants According to Marital Status **eTable 3.** Association of Marital Status With Risk of All-Cause Mortality in Asian Populations **eTable 4.** All-Cause Mortality Associated With Marital Status by Birth Years and Countries in Asian Populations
