# Supplementary material for: Association of Marital Status With Total and Cause-Specific Mortality in Asia
Source: JAMA Netw Open. 2022 May 31;5(5):e2214181. doi: 10.1001/jamanetworkopen.2022.14181 (PMC9157263; doi:10.1001/jamanetworkopen.2022.14181)
Supplement: Supplement. — eAppendix. Supplemental Methods eTable 1. Descriptions and Basic Characteristics of Participating Cohorts eTable 2. Baseline Characteristics for 623,140 Participants According to Marital Status eTable 3. Association of Marital Status With Risk of All-Cause Mortality in Asian Populations eTable 4. All-Cause Mortality Associated With Marital Status by Birth Years and Countries in Asian Populations [file jamanetwopen-e2214181-s001.pdf]

Supplemental Online Content

Leung CY, Huang HL, Abe SK, et al. Association of marital status with total and cause-specific mortality in Asia. *JAMA Netw Open*. 2022;5(5):e2214181. doi:10.1001/jamanetworkopen.2022.14181

eAppendix. Supplemental Methods
eTable 1. Descriptions and Basic Characteristics of Participating Cohorts
eTable 2. Baseline Characteristics for 623,140 Participants According to Marital Status
eTable 3. Association of Marital Status With Risk of All-Cause Mortality in Asian Populations
eTable 4. All-Cause Mortality Associated With Marital Status by Birth Years and Countries in Asian Populations

This supplemental material has been provided by the authors to give readers additional information about their work.

© 2022 Leung CY et al. *JAMA Network Open.*

eAppendix. Supplemental Methods

Assessment of exposure

Analyses for subcategories of unmarried status (single, separated, widowed, and divorced) were performed where data were available. For JPHC1 and JPHC2, living arrangement with spouse was used as a proxy for being married, all others were categorized as not married. There were three unmarried status (single, widowed, and divorced) assessed in JACC and SCS, whereas the unmarried status in CVDFACTS were single, separated, and widowed. In two studies (RERF and SCHS/SGC), separated and divorced were combined into one category in the questionnaire; therefore, they were excluded from the analysis for separated and divorced. Miyagi and Ohsaki studies were not included for the analysis of separated, widowed, and divorced, since these three categories were merged into one in the questionnaires.

Outcome ascertainment

The causes of death were classified using the International Classification of Diseases, 9th or 10th Revision (ICD–9 or ICD–10): cancer (ICD–9 codes 140–208, ICD–10 codes C00 to C97), circulatory system diseases (ICD–9 codes 390–459, ICD–10 codes I00 to I99), coronary heart disease (ICD–9 codes 410–414, ICD–10 codes I20 to I25), cerebrovascular disease (ICD–9 codes 430–438, ICD–10 codes I60 to I69), respiratory diseases (ICD–9 codes 460–519, ICD–10 codes J00–J99), and external causes (ICD–9 codes 800–999, ICD–10 codes S00–T98).

© 2022 Leung CY et al. *JAMA Network Open.*

eTable 1. Descriptions and Basic Characteristics of Participating Cohorts

Cohort	Sample / Representativeness	Study entry	Collection of covariates	Baseline age, mean (SD)	Men /Women, No. (%)	Baseline health condition, No. (%)	Ref
**China, Mainland**							
SCS	A population-based cohort. Data were collected from the general population in four communities in urban Shanghai.	1986-1989	Structured questionnaire	55.9 (5.7)	16, 751 (100) / 0 (0)	Healthy: 12,363 (73.8), HTN / DM: 4,256 (25.4), Cancer / CVD / CHD: 132 (0.8)	JAMA. 1996;275(21):1646-50.
SMHS	A population-based cohort. Data were collected from the general population in eight communities in urban Shanghai.	2001-2006	Self-administered questionnaire survey	55.7 (9.7)	53,205 (100) / 0 (0)	Healthy: 34,358 (64.6), HTN / DM: 14,411 (27.1) Cancer / CVD / CHD: 4,436 (8.3)	Int J Epidemiol. 2015;44(3)810-8.
SWHS	A population-based cohort. Data were collected from the general population in seven communities in urban Shanghai.	1996-2000	Self-administered questionnaire survey	52.6 (9.1)	0 (0) / 74,743 (100)	Healthy: 52,566 (70.3), HTN / DM: 14,702 (19.7), Cancer / CVD / CHD: 7,475 (10)	Am J Epidemiol. 2005;162(11):1123-31.
**Japan**							
JACC	A population-based cohort including data collected from the general population in 45 areas.	1988-1990	Self-administered questionnaire survey	57.1 (10.0)	31,337 (41.8) / 43,652 (58.2)	Healthy: 55,436 (73.9), HTN / DM: 15,528 (20.7), Cancer / CVD / CHD: 4,025 (5.4)	J Epidemiol. 2013;23(3):227-32.
JPHC1	A population-based cohort. Data were collected from five public health center areas across Japan.	1990-1992	Self-administered questionnaire survey	49.6 (6.0)	20,352 (47.8) / 22,235 (52.2)	Healthy: 33,585 (78.9), HTN / DM: 7,487 (17.6), Cancer / CVD / CHD: 1,515 (3.6)	J Epidemiol. 2001;11(6 Suppl):S3-7.
JPHC2	A population-based cohort. Data were collected from six public health center areas across Japan.	1992-1995	Self-administered questionnaire survey	54.2 (8.8)	26,407 (47.3) / 29,434 (52.7)	Healthy: 40,707 (72.9), HTN / DM: 12,878 (23.1), Cancer / CVD / CHD: 2,256 (4.04)	J Epidemiol. 2001;11(6 Suppl):S3-7.
Miyagi	A population-based cohort including data collected from 14 municipalities of Miyagi Prefecture.	1990	Self-administered questionnaire survey	51.9 (7.5)	18,919 (50.8) / 19,002 (50.1)	Healthy: 28,892 (76.2), HTN / DM: 7,859 (20.7), Cancer / CVD / CHD: 1,170 (3.1)	J Epidemiol. 2004;14 Suppl 1(Suppl I):S2-6.
Ohsaki	A cohort study on all National Health Insurance beneficiaries, aged 40-79 years, who lived in the catchment area of the Ohsaki Public Health Center, Miyagi, Japan	1995	Self-administered questionnaire survey	58.0 (10.8)	14,609 (54.2) / 12,341 (45.8)	Healthy: 18,120 (67.2), HTN / DM: 6,754 (25.1), Cancer / CVD / CHD: 2,076 (7.7)	J Epidemiol. 1998;8(5):258-63.
RERF	A cohort including 120,000 atomic bomb survivors and matched reesidents who were	1963-1993	Self-administered questionnaire survey	52.1 (13.6)	18,691 (39.6) / 28,509 (60.4)	Healthy: 34,740 (73.6), HTN / DM: 12,460 (26.4)	Radiat Res. 1962;16:253-80.

© 2022 Leung CY et al. *JAMA Network Open.*

	not in Hiroshima or Nagasaki at the time of bombing.						
Takayama	A population-based cohort including data collected from Takayama residents aged 35 years or above.	1992	Self-administered questionnaire survey	55.8 (12.8)	13,961 (45.7) / 16,613 (54.3)	Healthy: 22,345 (73.1), HTN / DM: 5,917 (19.4), Cancer / CVD / CHD: 2,312 (7.6)	Jpn J Clin Oncol. 1999;29(1):38-44.
**Korea, Republic of**							
KMCC	A population-based cohort including participants who were voluntary participants in a cancer screening survey in 19 sites in South Korea.	1993-2004	Trained interviewers administered questionnaires	53.9 (14.5)	7,718 (39.9) / 11,619 (60.1)	Healthy: 15,794 (81.7), HTN / DM: 2,542 (13.2), Cancer / CVD / CHD: 1,001 (5.2)	Asian Pacific J Cancer Prev, 3, 85-92.
KNCC	A cohort including participants who were enrolled for health check-ups at the Center for Cancer Prevention and Detection at the National Cancer Center in South Korea	2001-2015	Trained interviewers administered questionnaires	49.8 (9.2)	19,113 (50.8) / 18,525 (49.2)	Healthy: 28,611 (76.0), HTN / DM: 6,781 (18.0), Cancer / CVD / CHD: 2,246 (6.0)	Epidemiol Health. 2014; 36: e2014013.
Seoul-Male	A cohort including men living in or near Seoul, who were beneficiaries of the Korea Medical Insurance Corporation.	1992-1993	Self-administered questionnaire survey	49.2 (5.2)	13,957 (100) / 0 (0)	Healthy: 12,400 (88.8), HTN / DM: 1,471 (10.5), Cancer / CVD / CHD: 86 (0.6)	Journal of Preventive Medicine and Public Health 2012; 45(1): 14-20.
**Singapore**							
SCHS (SGC)	A population-based cohort. Data were collected from permanent residents or citizens of Singapore who resided in government-built housing estates.	1993-1999	Self-administered questionnaire survey	56.4 (8.0)	27,593 (44.0) / 35,065 (56.0)	Healthy: 42,557 (67.9), HTN / DM: 15,118 (24.1), Cancer / CVD / CHD: 4,983 (8.0)	Nutr Cancer. 2001;39(2):187-95.
**Taiwan**							
CBCSP	A cohort including residents in seven townships in Taiwan.	1991-1992	Trained public health nurses administered questionnaires	47.3 (10.0)	11,883 (50.2) / 11,787 (49.8)	Healthy: 21,391 (90.4), HTN / DM: 1,644 (7.0), Cancer / CVD / CHD: 635 (2.7)	N Engl J Med. 2002;347(3):168-74.
CVDFACTS	A cohort randomly selecting residents of ten villages in two townships in Taiwan (Chu-Dung and Pu-Tze).	1990-1993	Trained interviewers administered questionnaires	47.5 (15.6)	2,247 (43.9) / 2,872 (56.1)	Healthy: 4,267 (83.4), HTN / DM: 672 (13.1), Cancer / CVD / CHD: 180 (3.5)	Stroke. 2009;40(5):1578-84.

Abbreviations: No., number; CVD, cerebrovascular disease; CHD, coronary heart disease; DM, diabetes mellitus; HTN, hypertension; SCS, Shanghai Cohort Study; SMHS, Shanghai Men's Health Study; SWHS, Shanghai Women's Health Study; JACC, Japan Collaborative Cohort Study; JPHC, Japan Public Health Center-based prospective Study; Miyagi, Miyagi Cohort Study; Ohsaki, Ohsaki National Health Insurance Cohort Study; RERF, Life Span Study Cohort-Radiation Effects Research Foundation; Takayama, Takayama Study; KMCC, Korean Multi-center Cancer Cohort Study; KNCC, Korea National Cancer Center Cohort; Seoul-Male, Seoul Male Cancer Cohort; SCHS, Singapore Chinese Health Study; CBCSP, Community-based Cancer Screening Project; CVDFACTS, Cardiovascular Diseases Risk Factor two-Township Study.

© 2022 Leung CY et al. *JAMA Network Open.*

eTable 2. Baseline Characteristics for 623,140 Participants According to Marital Status

Characteristic	Married	Single	Separated	Divorced	Widowed
Participants, No. (%)	538,377 (86.4)	14,454 (2.3)	1,347 (0.2)	35,212 (5.7)	5,674 (0.9)
Men, No. (%)	268,975 (50.0)	7,452 (51.6)	228 (16.9)	6,437 (18.3)	1,792 (31.6)
Women, No. (%)	269,402 (50.0)	7,002 (48.4)	1,119 (83.1)	28,775 (81.7)	3,8822 (68.4)
Age, mean (SD), y	53.3 (9.7)	46.7 (13.8)	52.4 (9.9)	62.2 (11.1)	51.6 (9.7)
**Smoking status**					
Never, No. (%)	311,216 (57.8)	7,973 (55.2)	1,043 (77.4)	25,184 (71.5)	3,238 (57.1)
Former, No. (%)	57,654 (10.7)	1,038 (7.2)	79 (5.9)	2,037 (5.8)	448 (7.9)
Current, No. (%)	153,776 (28.6)	4,960 (34.3)	217 (16.1)	6,407 (18.2)	1,760 (31.0)
Missing, No. (%)	15,731 (2.9)	483 (3.3)	8 (0.6)	1,584 (4.5)	228 (4.0)
**Alcohol intake**					
Non-drinker, No. (%)	327,087 (60.8)	8,629 (59.7)	1,042 (77.4)	26,033 (73.9)	3,207 (56.5)
Drinker, No. (%)	200,711 (37.3)	5,516 (38.2)	299 (22.2)	8,614 (24.5)	2,355 (41.5)
Missing, No. (%)	10,579 (2.0)	309 (2.1)	6 (0.5)	565 (1.6)	112 (2.0)
**Physical activity**					
None or almost none, No. (%)	181,437 (33.7)	2,448 (16.9)	580 (43.1)	7,937 (22.5)	2,175 (38.3)
Low, No. (%)	62,481 (11.6)	1,880 (13.0)	199 (14.8)	3,628 (10.3)	1,002 (17.7)
Intermediate, No. (%)	139,066 (25.8)	4,969 (34.4)	200 (14.9)	9,973 (28.3)	792 (14.0)
High, No. (%)	36,654 (6.8)	484 (3.35)	168 (12.5)	3,147 (8.9)	358 (6.3)
Missing, No. (%)	118,739 (22.1)	4,673 (32.3)	200 (14.9)	10,527 (29.9)	1,347 (23.7)
**Education**					
No formal or primary education, No. (%)	193,501 (39.6)	5,649 (39.3)	337 (28.0)	23,495 (66.7)	1,780 (31.4)
Secondary education, No. (%)	158,878 (32.5)	4,990 (34.7)	509 (37.8)	6,690 (19.0)	2,123 (37.4)
Trade or technical education, No. (%)	56,633 (11.6)	1,171 (8.1)	247 (18.3)	1,412 (4.0)	775 (13.7)
University education or above, No. (%)	62,522 (12.8)	2,088 (14.5)	198 (14.7)	1,589 (4.5)	740 (13.1)
Missing, No. (%)	17,211 (3.5)	483 (3.4)	16 (1.2)	2,022 (5.7)	252 (4.4)

Abbreviations: No., number; CI, confidence intervals.

© 2022 Leung CY et al. *JAMA Network Open.*

eTable 3. Association of Marital Status With Risk of All-Cause Mortality in Asian Populations

	HR (95% CI) [a]					
	**Married (n=538,377)**	**Not married (n=84,763)**	**Single (n=13,159)**	**Separated (n=1,168)**	**Divorced (n=4,671)**	**Widowed (n=33,997)**
**Multivariable model**						
Death no.	100,249	23,015	3,072	164	641	11,651
All-cause mortality	1 [ref]	1.15 (1.07-1.24)	1.62 (1.41-1.86)	1.35 (1.13-1.61)	1.38 (1.13-1.69)	1.09 (1.04-1.13)
**Excluding first 5 years of follow-up**						
Death no.	85,101	19,129	2,536	136	502	9,466
All-cause mortality	1 [ref]	1.14 (1.05-1.22)	1.59 (1.38-1.83)	1.42 (1.18-1.71)	1.28 (1.07-1.54)	1.08 (1.04-1.13)

Abbreviations: HR, hazard ratio; CI, confidence intervals; no., number.

[a] Multivariable Cox regression model adjusted for sex, age at baseline (year, continuous), smoking status (never, former, current, and missing), education (no formal education or primary education, secondary education, trade or technical education, university education or above, and missing), physical activity (none or almost none, low, intermediate, high, and missing), alcohol intake (non-drinker, drinker, and missing), and baseline health conditions (stroke, coronary heart disease, cancer, hypertension, and diabetes mellitus).

© 2022 Leung CY et al. *JAMA Network Open.*

eTable 4. All-Cause Mortality Associated With Marital Status by Birth Years and Countries in Asian Populations

Characteristic	Married	Not married HR (95%CI)	*P* for interaction	Single HR (95%CI)	*P* for interaction	Separated HR (95%CI)	*P* for interaction	Divorced HR (95%CI)	*P* for interaction	Widowed HR (95%CI)	*P* for interaction
**Birth year [a]**			<0.001		0.007		0.06		0.13		0.12
Death no.	84,626	20,562		744		53		168		419	
Before 1940	1 [ref]	1.10 (1.04-1.17)		1.44 (1.24-1.68)		1.22 (1.01-1.48)		1.23 (1.01-1.51)		1.08 (1.04-1.12)	
Death no.	15,623	2,453		764		75		192		7,416	
In or after 1940	1 [ref]	1.46 (1.26-1.69)		1.96 (1.66-2.32)		2.03 (1.32-3.12)		1.60 (1.22-2.10)		1.18 (1.04-1.35)	
**Country [a]**			0.11		0.19		0.06		0.54		0.21
Death no.	19,816	2,381		258		70		265		1,788	
Mainland China	1 [ref]	1.32 (1.10-1.60)		2.14 (1.80-2.54)		1.14 (0.90-1.44)		1.39 (0.99-1.96)		1.11 (1.06-1.17)	
Death no.	63,269	16,032		2,073		37		320		6,349	
Japan	1 [ref]	1.08 (1.00-1.17)		1.44 (1.20-1.73)		1.51 (1.09-2.09)		1.18 (0.96-1.46)		1.06 (0.99-1.13)	
Death no.	3,994	1,037		46		37		38		916	
Korea	1 [ref]	1.18 (1.09-1.28)		1.67 (1.12-2.50)		1.31 (0.95-1.81)		1.98 (1.33-2.96)		0.87 (0.55-1.38)	
Death no.	10,258	2,980		497		--		--		2,249	
Singapore	1 [ref]	1.16 (1.11-1.21)		1.39 (1.27-1.52)		--		--		1.11 (1.06-1.17)	
Death no.	2,912	585		198		20		18		349	
Taiwan	1 [ref]	1.18 (0.75-1.87)		1.44 (1.20-1.73)		1.76 (1.13-2.75)		1.06 (0.67-1.70)		1.03 (0.74-1.44)	

Abbreviations: HR, hazard ratio; CI, confidence intervals; no., number.

[a] Multivariable Cox regression model adjusted for gender, age at baseline (year, continuous), smoking status (never, former, current, and missing), education (no formal education or primary education, secondary education, trade or technical education, university education or above, and missing), physical activity (none or almost none, low, intermediate, high, and missing), alcohol intake (non-drinker, drinker, and missing), and baseline health conditions (stroke, coronary heart disease, cancer, hypertension, and diabetes mellitus).

© 2022 Leung CY et al. *JAMA Network Open.*
